# Characterization of circulating molecules and activities in plasma of patients after allogeneic and autologous intraoral bone grafting procedures: a prospective randomized controlled clinical trial in humans

**DOI:** 10.1186/s12903-021-02036-7

**Published:** 2022-01-30

**Authors:** Önder Solakoglu, Bettina Steinbach, Werner Götz, Guido Heydecke, Heidi Schwarzenbach

**Affiliations:** 1 Dental Department, Center for Dental and Oral Medicine, University Medical Center Hamburg-Eppendorf, Martinistr. 52, 20246 Hamburg, Germany; 2 Practice Limited to Periodontology and Implant Dentistry, Hamburg, Germany; 3 Department of Tumor Biology, University Medical Center Hamburg-Eppendorf, Hamburg, Germany; 4 Laboratory for Oral Biologic Basic Science, Department of Orthodontics, University of Bonn, Bonn, Germany

**Keywords:** Allogeneic bone grafts, Autologous bone grafts, Cf DNA, microRNAs, Exosome, Caspase, Plasma

## Abstract

**Background:**

The objective was to assess whether intraoral bone augmentation procedures have an impact on the patient’s plasma levels of circulating nucleic acids, exosomes, miRNA levels and caspase activities. The null hypothesis was tested, that no significant differences between the two groups will be found.

**Methods:**

In this prospective randomized controlled clinical trial 35 systemically healthy non-smoking participants were randomly allocated using sealed envelopes by a blinded clinician not involved in the clinical setting. Plasma samples were collected preoperatively and 3 times postoperatively (immediately, 5 weeks and 4 months postoperatively). The test group consisted of twenty-five patients who received allogeneic bone grafting material and the control group of ten patients who received autologous bone grafts. Levels of cell-free DNA (cfDNA) and microRNAs (miR-21, miR-27a, miR-218) were quantified by real-time PCR, caspase activities and exosome concentrations were determined by ELISA.

**Results:**

Statistical evaluation reveled a significantly higher exosome level before surgery (p = 0.013) and the first postsurgical sample (p = 0.017) in the control group compared to the test group. The levels of miR-27a and miR-218 significantly differed between the plasma samples before surgery and after surgery in both groups. The levels of miR-21 only significantly differed between the pre- and postsurgical plasma samples in the test group, but not in the control group. All patients completed the study, no adverse events were recorded.

**Conclusions:**

Our data show the diagnostic potential of the plasma levels of miR-27a, miR-218 and miR-21 in detecting changes in bone metabolism after alveolar bone augmentation. Our very promising results indicate that there might be a high diagnostic potential in evaluating the plasma levels of the before mentioned miRNAs in order to detect bone resorption activities before they become clinically relevant.

*Trial registration* Ethical commission of the Ärztekammer Hamburg, Germany (PV5211) on 11/03/2016 as well as by the German Registry of Clinical Studies (DRKS 00,013,010) on 30/07/2018 (http://apps.who.int/trialsearch/).

**Supplementary Information:**

The online version contains supplementary material available at 10.1186/s12903-021-02036-7.

## Introduction

### Background

Within the last decades the replacement of missing teeth with dental implants became a predictable and safe treatment modality [1]. However, the loss of natural teeth is in the majority of cases associated with loss of alveolar bone due to traumatic events or inflammatory diseases like periodontal disease [2]. Despite the well described technique of alveolar ridge preservation following tooth extraction, which was investigated in multiple prospective and controlled clinical trials, demonstrating a significant decrease in the need for advanced regenerative bone augmentation procedures [3–5] it is nevertheless very common that bone augmentation procedures become necessary in order to vertically and horizontally rebuilt the previously lost bone volume prior to implant placement [6].

Different bone grafting materials for intraoral bone augmentation are widely used in oral surgery procedures [7]. Autologous bone is still considered the gold standard, however, its intraoral availability is limited and associated with a harvesting site. Therefore, the use of bone grafting materials became commonly accepted in order to reduce intra- and extraoral harvesting of autologous bone and to reduce surgical morbidity [8]. Among these bone substitutes allogeneic bone grafting materials are widely used. Several investigations regarding the safety and efficiency of those materials are available [9–12]. During intraoral bone augmentation procedures, patients who received bone grafts can undergo cellular stress or tissue injury resulting in inflammatory responses. These pathogenic processes are initiated, sustained and propagated by a coordinated expression of numerous genes and lead to tissue remodeling as well as provoke the release of circulating nucleic acids into the blood circulation. Since the levels of circulating cell-free DNA (cfDNA) and microRNAs (miRNAs) are usually early changed in the blood circulation after onset of such complaints, their measurements may reflect disease pattern and course along with inflammatory processes [13]. Shao et al. refer to p53 as a possible early marker and show a schematic presentation of pathways for cell-free DNA [14].

The mechanism of the release of nucleic acids into the blood circulation occurs either by proliferating cells that actively secrete exosomes or dying cells, such as apoptotic and necrotic cells [15]. Caspases which belong to the cysteine proteases are involved in the apoptotic cascade. Their increase is a further essential aspect of pathogenesis [16]. During apoptosis, caspase‐activated endonucleases induce the fragmentation of DNA into nucleosomal units of about 200 bp and multiple of them. This DNA is then packed in apoptotic blebs that are subsequently engulfed by neighboring macrophages and released into the blood circulation [17].

In order to investigate whether cfDNA levels in the bloodstream reveal the acceptance of a surgical intervention, housekeeping genes or repetitive sequences are commonly quantified as target genes in a PCR. In the current study, we amplified Alu sequences which are most frequent, repetitive DNA elements interspersed throughout the whole genome. These transposable elements are one of major sources to cause genomic instability through various mechanisms including de novo insertion, insertion-mediated genomic deletion, and recombination-associated genomic deletion [18]. Calculation of the ratio between longer and shorter Alu-DNA fragments, namely the cfDNA integrity, reflects the different cell deaths. Shorter fragments of < 200 bp are considered to be derived from apoptotic cells and larger ones of > 200 bp to be derived from necrosis.

Exosomes are small nanoparticles (50–120 nm) derived from cellular endosomes, and actively secreted by exocytosis by all cells into the microenvironment of the cell and body fluids. Although, at first, they were considered as cellular waste, nowadays it is known that they have pleiotropic physiological and pathological functions. Exosomes regulate an important event, namely the intercellular communication. They can transport their bioactive cargo from cell to cell, and in such a way they manipulate the behavior of the recipient cell [19, 20]. It is evident that various benign and malignant diseases cause elevated levels of exosomes in the blood circulation [21].

Besides DNA, RNA, proteins and lipids, exosomes also contain miRNAs. MiRNAs are small regulatory, non-coding RNA molecules and consist of approximately 22 nucleotides. As one of the largest gene families, miRNAs account for about 1% of the human genome, and are supposed to regulate more than 50% of all protein-coding genes [22]. Their main function is the modulation of the activity of specific mRNA molecules. By their sequence-specific binding to complementary sequences in the 3′-untranslational region (3′UTR) of their target mRNAs, they inhibit the translation of their target into polypeptides or degrade them. Thus, they are able to alter the protein pattern of a cell [23]. They are involved in the regulation of numerous signaling pathways and cellular processes, e.g. apoptosis, immunoreactions, hematopoietic cell differentiation and metabolism [24]. Cell-free miRNAs circulate stable within the blood, and their levels reflect various benign and malignant diseases [25, 26]. For our current study, we selected miR-21, miR-27a and miR-218 from the wide variety of miRNAs, since they can participate in different diseases and their importance in inflammation and osteogenesis has been reported [27–29]. Since the equilibrium of bone apposition and bone resorption is fundamental for the long-term stability of dental implants as well as the initiation and progression of periodontal disease, there is an urgent need for a minimally invasive blood test to identify the presence and the activity of different cell-regulating molecules.

In our recently published study, we detected increased levels of cfDNA in allograft and autograft patients immediately postoperatively which may be caused by surgical procedures [30]. These investigations prompted us to extend our study on circulating components in these patients that may be released by inflammatory and osteogeneic processes [16] or oral [31] and periodontal diseases [32].

### Aim

The aim of the present study was to test the null hypothesis that no differences exist between the pre-and postoperative levels of circulating nucleic acids, exosomes, miRNA levels and caspase activities in plasma of patients before and after allogeneic and autologous intraoral bone grafting procedures. Those levels were measured in plasma in order to avoid assessment of genomic DNA in cells of the blood and too fragmented DNA in saliva. As far as we know, this is the first study to measure changes in the levels and activities of different molecules circulating in plasma of patients undergoing intraoral bone grafting procedures. Potential changes of those levels may indicate inflammatory processes in the patients and therefore the clinical outcome of these surgical procedures. This is of particular clinical relevance in order to improve these products and procedures for the benefit of the patients.

## Materials and methods

### Study design, participants, and collection of plasma samples

The publication was performed and written according to the CONSORT Guidelines [33] for randomized controlled clinical trials. Blood samples were collected from 25 women who received allogeneic bone grafting material (Maxgraft® Allograft Spongiosa Particle (Botiss Company, Berlin, Germany, part of Straumann Group, Basel, Switzerland)) from male donors (allograft patients, test group) and from 10 women who were treated with autologous graft (autograft patients, control group) for lateral ridge augmentation procedures recruited and treated in a single periodontal office in Hamburg, Germany, between September 2018 to February 2019. All patients were systemically healthy non-smokers and were randomly assigned to one of the parallel groups by a blinded clinician not involved in this study and not involved in the periodontal office by drawing sealed envelopes. Blood samples were collected on the day of surgery preoperatively, immediately postoperatively, at 5 weeks and 4 months after surgery. Plasma was prepared and stored in liquid nitrogen. The mean age of allograft patients was 58 years (range 39–78), and autograft patients were 55 years (range 32–76). The collection of the blood samples and experiments were performed in compliance with the World Medical Association Declaration of Helsinki (version 2008) and were approved by an ethics committee (Hamburg Medical Association, Germany, no. PV5211) and the study was registered with the German Register for Clinical Trials (DRKS No. 00013010). All patients gave their informed consent and all patients completed the study successfully and were available for follow-up visits. No adverse events were recorded.

### Surgical procedure

**Fig. 1:**
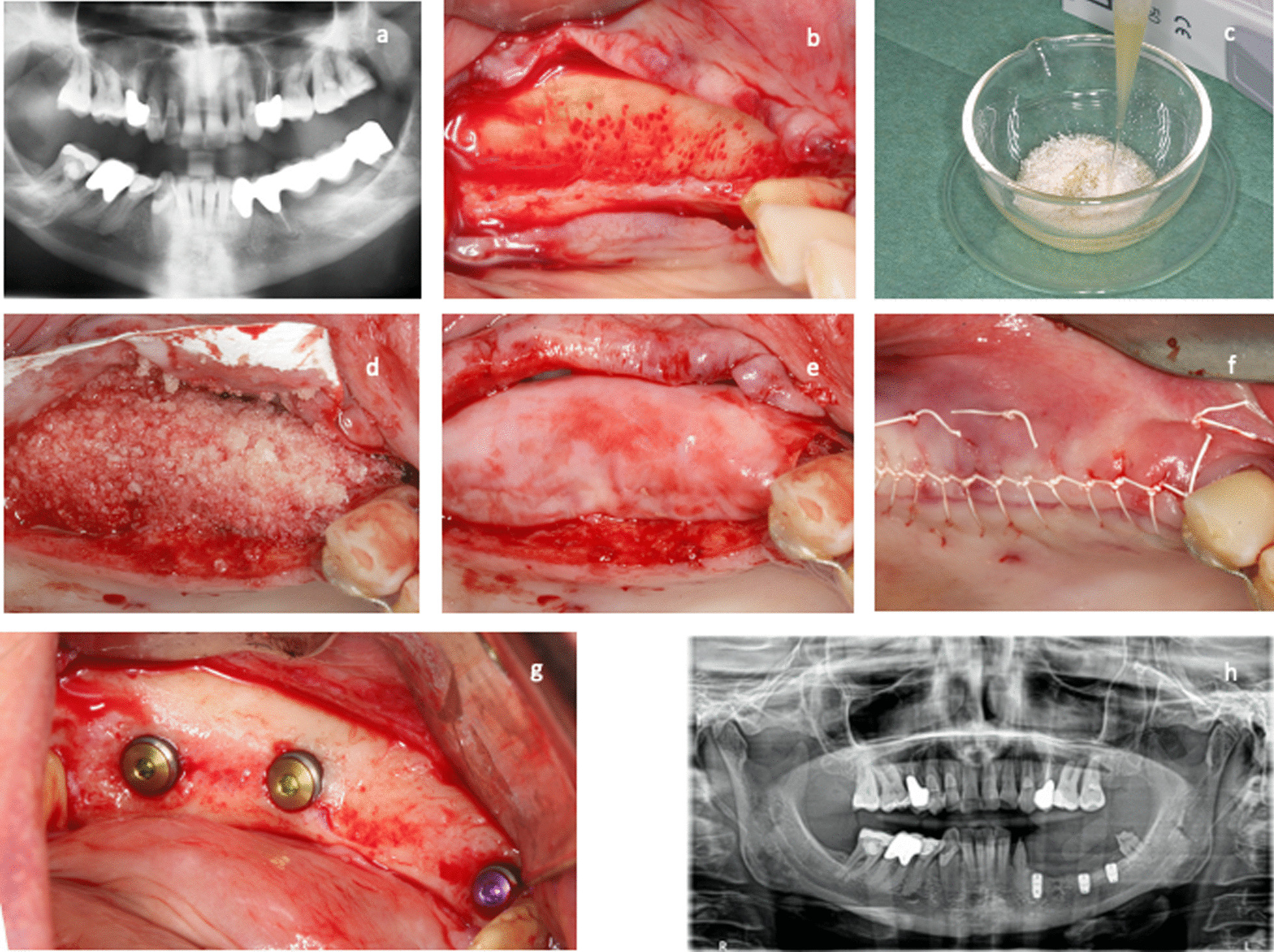
**a**–**h** Surgical procedure of the lateral ridge augmentation technique. In the initial radiograph **a** a ridge deficiency of the alveolar ridge in the third quadrant is visible. **b** shows the clinical appearance of the alveolar ridge defect after reflection of a mucoperiosteal flap. **c** Bone allograft material (Maxgraft, Botiss company) which is rehydrated and used for lateral augmentation of the alveolar crest (**d**) and covered with a pericardium membrane (**e**). The flap was sutured with a 5.0 Gore Tex suture in order to achieve primary closure (**f**). The clinical view after 4 months of healing is visible in **g**, at time of implant insertion. The postoperative panoramic radiograph after implant insertion is shown in **h**

Bone grafting for alveolar ridge augmentation were performed under local anesthesia using Ultracain-DS Forte (Sanofi-Aventis, Frankfurt/Main, Germany). After deflection of a mucoperiosteal flap a cortical perforation was done and allogeneic bone grafting material (test group) or autogenous bone (control group) was inserted. The bone grafts were covered with a collagen membrane (Jason Membrane, Botiss Company) for guided bone regeneration, according to the manufacturer’s recommendations (Botiss Company, Berlin, Germany, part of Straumann Group, Basel, Switzerland). A periosteal releasing incision of the mucoperiosteal flap was performed in order to mobilize the flap for a tension-free primary closure of the surgical site. Flap-fixation was performed using a horizontal and vertical mattress suture with 5.0 Goretex filaments (W. L. Gore & Associates GmbH, Putzbrunn, Germany, Fig. 1). A 2.0% chlorhexidine rinsing solution was administered for post-operative oral hygiene. Post-operative appointments were scheduled after 1–2 days, 2, 6, and 12 weeks. Sutures were removed 2 weeks after augmentation. Two-dimensional radiographs, using the parralleling technique with a Rinn holder (Dentsply-Rinn, 1301 Smile Way, York, PA 17404, USA) were taken immediately following the bone augmentation procedure.

### Extraction of circulating cfDNA from plasma samples

Using PaxGene Blood ccf tubes (Qiagen, Hilden, Germany), blood was collected preoperatively and postoperatively (collected on the same day) from 25 allograft and 10 autograft patients. Plasma was prepared by 2 centrifugation steps at 2670 and 10,000*g*, each for 10 min, and tested for hemolysis. The samples were not hemolytic [30].

Circulating cfDNA was extracted from a total of 120 plasma samples using the QIAamp Circulating Nucleic Acid kit (Qiagen) according to the manufacturer’s instructions. Briefly, 3 mL plasma was mixed with 2.4 ml ACL buffer (containing 0.2 μg/μL carrier RNA) plus 300 μL of proteinase K for 30 s, and incubated at 60 °C for 30 min. After addition of 5.4 mL ACB buffer and incubation on ice for 5 min., the lysate was drawn through a Qiamp Mini column. The column was washed with 600 μL ACW1 buffer, 750 μL ACW2 buffer and 700 μL 100% ethanol. CfDNA was precipitated from the column by 30 μL AVE buffer.

The quantity of the extracted cfDNA was determined spectrophotometrically using the Qubit dsDNA HS Assay kit (Thermo Fisher Scientific). Briefly, 2 μL cfDNA was diluted in 198 μL HS Reagent solved in HS Buffer (1:200), incubated for 3 min at room temperature and measured on the Qubit 2.0 Fluorometer (Thermo Fisher Scientific). The fluorometer was calibrated with Standard 1 and 2 of the kit. We repeated the measurement of each sample once and received the same data.

### Quantitative PCR of Alu elements

In a 10-μl reaction, 1 μl of 1:5 diluted DNA was mixed with 5 μl SYBR Green qPCR Mastermix (Qiagen) and the following 0.25 μl 10 mM Alu115 and Alu247 primer sets:

- Alu115 primer forward: 5′-CCTGAGGTCAGGAGTTCGAG-3′
- reverse: 5′-CCCGAGTAGCTGGGATTACA-3′
- Alu247 primer forward: 5′-GTGGCTCACGCCTGTAATC-3′
- reverse: 5′-CAGGCTGGAGTGCAGTGG-3′

The Alu115 and Alu247 primer sets amplified PCR products at sizes of 115 and 247 bp, respectively. A negative control without any template was also performed on each plate. The reactions were run on an MJ Research PTC-200 Peltier Thermal Cycler (Global Medical Instrumentation, Ramsey, MN, USA). The following real-time PCR program was carried out: 1 cycle at 95 °C for 15 min; 40 cycles at 95 °C for 30 s, 64 °C for 30 s and 72 °C for 30 s. Subsequently, a melting curve analysis was carried out as followed: 95 °C for 15 s, 60 °C for 15 s and heating up to 95 °C in 20 min and holding the temperature for 15 s. Mean values were calculated from triplicate reactions.

CfDNA integrity was calculated as a ratio of concentrations of long to short cfDNA fragments of Alu elements (Alu247/Alu115).

### Caspase assay

For measurements of the activity of caspase 3 and 7, the Caspase Glo®3/7 assay (Promega, Mannheim, Germany) was carried out. The Caspase Glo®3/7 assay is based on the cleavage of the DEVD sequence of a luminogenic substrate by the caspases 3 and 7 which results in a luminescent signal. To draw a standard curve from 5 × 10^−3^ down to 5 × 10^−6^, dilution steps in a ratio 1:2 were carried. Briefly, 1 μl caspase solution (100 U/μl, Enzo Life Sciences AG, Lausen, Switzerland) was twice diluted in 100 μl enzyme dilution buffer (50 mM HEPES, pH 7.4, 100 mM sodium chloride, 0.5% CHAPS, 1 mM EDTA, 10% glycerol and 10 mM dithiothreitol). Then, this 0.01 U/μl caspase solution was 11 times serially diluted in a ratio of 1:2. In each case, 50 μl of these standards as well as the plasma samples were mixed with 50 μl Caspase-Glow reagent (Promega) and incubated on a shaker at 200 rpm for 1 h. On a 96 well-plate, the samples were measured on a Glomax Luminometer 20/20 (Thermo Fisher Scientific) for 1 s. A blank reaction was used to measure background luminescence.

### Exosome assay

For measurements of the exosome concentrations, the ExoQuantTM Overall Exosome Capture and Quantification assay, a double sandwich ELISA using an antibody against the exosomal marker CD63 (BioVision, Milpitas, California, USA), was carried out according to the manufacturer's instructions. For stabling the calibration curve, lyophilized exosomes were solved in 100 μl of deionized water and 100 μl of PBS to reach a final volume of 200 μl per vial. Standard dilutions were prepared directly in the strips by using the exosome solution to perform six two-fold serial dilutions with PBS. The standard concentrations were 50, 25, 12.5, 6.25, 3.125, 1.5625 and 0.78125 μg. Then, 100 μl plasma diluted to 1:1 in PBS was loaded on a 96-well plate pre-coated with proprietary pan-exosome antibodies enabling specific capture of exosomes. The plate was incubated at room temperature while shaking (2–3 rotations/s) for 30 min, and subsequently, at 4 °C overnight. After washing, 100 μl of mouse anti-human exosome Detection Antibody solution (diluted to 1:500 in Sample Buffer) was added to each well and incubated at room temperature while shaking for 15 min and subsequently at 4 °C for 2 h. After washing, 100 μl of rabbit anti-mouse IgG HRP-conjugated secondary antibody solution (diluted to 1:2000 in Sample Buffer) was added to each well and incubated at room temperature while shaking for 15 min and subsequently at 4 °C for 1 h. After washing, 100 μl of Substrate Chromogenic Solution was added to each well and incubated at room temperature in the dark for 10 min. The reaction was stopped by adding 100 μl of Stopping Solution to each well. For colorimetric detection, the absorbance at 450 and 570 nm was measured within 10 min on a Microplate reader (Tecan, Männerdorf, Switzerland).

### Extraction of miRNAs

MiRNAs were extracted in 100 μl plasma supplemented with 150 μl lysis buffer (Thermo Fisher Scientific, Vilnius, Lithuania) and 50 μl PBS (Life Technologies) by using the TaqMan miRNA ABC Purification Buffer Kit (Thermo Fisher Scientific). According to the manufacturer’s instructions, the miRNAs were bound to 80 μl anti-miR beads using the TaqMan miRNA ABC Purification Bead kit Human panel A (Thermo Fisher Scientific). To avoid technical variability, 2 μl of 1 nM synthetic non-human cel-miR-39 were added as an exogenous spike in control.

### Reverse transcription

Reverse transcription was carried out using a modified protocol of TaqMan MicroRNA Reverse Transcription kit (Thermo Fisher Scientific). The reaction contained 4.0 μl RT Primer Pool of miR-484, cel-miR-39 (reference miRNAs) and miR-21, miR-27a, miR-218 (miRNAs of interest) diluted in 1:100 Tris–EDTA, 0.2 μl dNTPs with 100 mM dTTP, 2.0 μl (50 U/μl) MultiScribe Reverse Transcriptase, 1 μl 10 × RT Buffer, 0.127 μl (20 U/μl) RNase Inhibitor and 2 μl miRNAs. The conversion of miRNAs into cDNA was carried out at 16 °C for 30 min, 42 °C for 30 min and 85 °C for 5 min on an MJ Research PTC-200 Peltier Thermal Cycler (Global Medical Instrumentation, Ramsey, Minnesota, USA).

### Preamplification of cDNA

To increase input cDNA, a pre-amplification step of cDNA was included. For TaqMan PCR analyses of miR-21, miR-27a and miR-218, cDNA of the reference miR-484 and cel-miR-39 was also preamplified. Here, 1 μl cDNA was preamplified in 5 μl TaqMan PreAmp Master Mix (Thermo Fisher Scientific) and 1.5 μl specific PreAmp primer pool. The reaction was run on a MJ Research PTC-200 Peltier Thermal Cycler (Global Medical Instrumentation): 1 cycle at 95 °C for 10 min, 55 °C for 2 min, 72 °C for 2 min; 16 cycles at 95 °C for 15 s, 60 °C for 4 min; and a final cycle 99.9 °C for 10 min. A negative control without any templates was included from the starting point of reverse transcription, too.

### TaqMan real time PCR analyses of miR-21, miR-27a and miR-218

For quantitative real-time PCR, the TaqMan miRNA assays (Thermo Fisher Scientific) for miR-484 and miR-39 (reference miRNAs), and miR-21, miR-27a miR-218 were used. In a 10 μl-reaction, 0.25 μl preamplified cDNA were mixed with 5 μl TaqMan Universal PCR Master Mix and 0.5 μl TaqMan MicroRNA Assay. Quantitative real-time PCR reaction was performed at 95 °C for 10 min. and in 40 cycles at 95 °C for 15 s and 60 °C for 60 s, on a C1000 Touch real-time PCR device (Bio-Rad, Hercules, California, USA).

### Data normalization of miRNA data

As there is no consensus on a reference miRNA for data normalization, we chose miR-484 for plasma quality control and cel-miR-39 for inter-individual variability of the efficiency of our procedures as an endogenous and exogenous reference gene, respectively, to normalize our miRNA data. The obtained data of the miRNA expression levels were calculated by the ΔCt method as follows: ΔCt = mean value Ct (reference cel-miR-39 + miR-484) − mean value Ct (miRNA of interest). The relative expression data were 2^(ΔCt)^ transformed in order to obtain normal distribution data. The confidence of 2^(ΔCt)^ data were verified by amplification curves and Ct confidence (0–1, whereby 1 refers to the highest confidence). Our data showed a Ct confidence of 0.95. Values below 0.95 were discarded.

### Statistical analysis

Statistical analyses were performed using the SPSS software package, version 24.0 (SPSS Inc. Chicago, IL, USA). Statistical differences in the measured data were calculated using ANOVA with Tukey's HSD test for all pairwise comparisons that correct for experiment-wise error rate. Two-sample comparisons were performed using Student's t-test for equal or unequal variance where appropriate. Due to the small size of the variables, the Holm-Bonferroni method was not employed for multiple test correction. Missing data were handled by pairwise deletion. A p-value ≤ 0.05 was considered as statistically significant. All p-values are two-sided (Additional file 2).

## Results

### Plasma cfDNA levels, cfDNA integrity, caspase activities and exosome concentrations

In our previous study, we investigated plasma samples exclusively collected from 25 female allograft patients who received male donors’ allogeneic material in order to investigate whether foreign DNA from the allogeneic bone grafting material migrates into the patients’ blood circulation. Our recent data [30] showed that at no point in time, Y-cfDNA was detected in the allograft patients’ plasma samples, indicating that no male DNA from the allogeneic bone material migrated into the patients’ bloodstream.

**Fig. 2:**
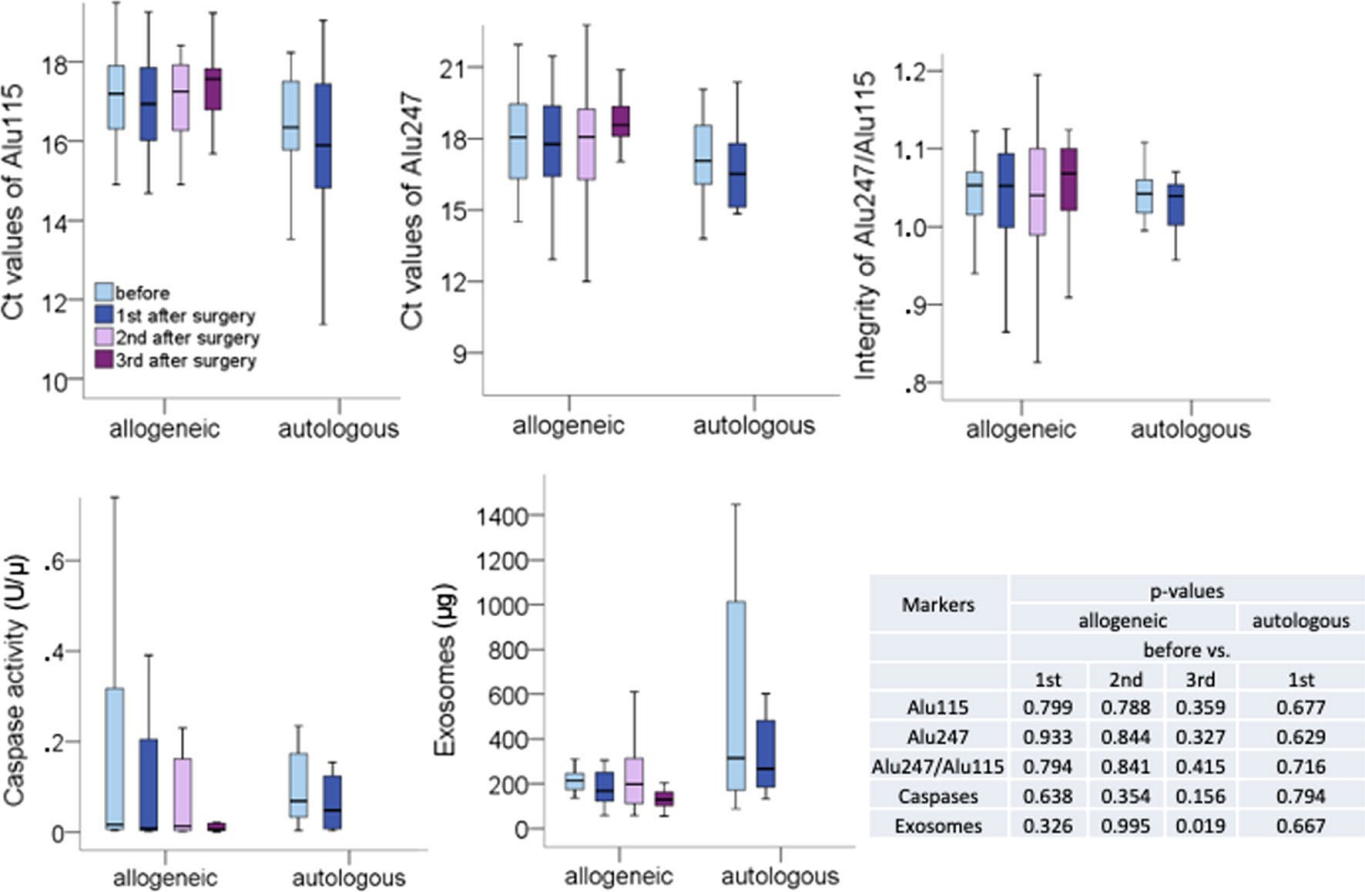
Plasma levels of cfDNA, cfDNA integrity, caspase activities and exosomes. The levels depicted as box plots were measured in one presurgical and three postsurgical plasma samples in each of 25 allogeneic and in one presurgical and one postsurgical plasma samples in each of 10 autolologous patients. The concentrations of Alu115 and Alu247 were quantified by real-time PCR, and the ratio of Alu247/Alu115 was calculated which served as cfDNA integrity. Caspase activities and exosome secretion were determined by specific ELISAs. Adjacent to the box plots, a table summarizes the p-values

In the present study we further investigated the plasma samples of these 25 patients (allogeneic test group) and compared the specific plasma levels to the 10 patients in the autogenous control group. The plasma levels of cfDNA were quantified by amplifying Alu sequences at lengths of 115 bp (Alu115) and 247 bp (Alu247). The cfDNA integrity which is an indicator of the extent of cfDNA fragmentation was determined by calculating the ratio of concentrations of long to short cfDNA fragments of Alu elements (Alu247/Alu115). In this regard, Alu115 is assumed to reflect the amounts of total cfDNA, and in particular apoptotic cfDNA in blood, whereas Alu247 is supposed to reflect necrosis and active DNA secretion. The statistical evaluation showed that there were no changes in the concentrations of Alu115 and Alu247, as well as in cfDNA integrity between pre- and postsurgical plasma samples in both patient cohorts. Although, in general, the allogeneic group showed higher cfDNA values than the autologous group, but they were not significant (Fig. 2 and Additional file 1).

In addition, the measurements of caspase activities and exosome levels did also not show any significant difference between pre- and postsurgical plasma samples, with one exception of the exosome levels between before surgery and the third sample 5 weeks after surgery (p = 0.019). However, this could be an outlier, due to the small number of allograft patients and the relative low significance (p = 0.019, Fig. 2). Moreover, further analyses of a fourth postsurgical sample taken 4 months following surgery showed that the exosome levels were not significant anymore (data not shown). A significantly higher exosome levels before surgery (p = 0.013) and the first postsurgical sample (p = 0.017) in the autologous group could be observed than in the corresponding samples of the allogeneic group. However, these significances may be caused by the high variance detected in the autologous group. In particular, it is striking that the variance of the levels of caspase activities and exosomes decreased in the third postsurgical sample in the allogeneic cohort, suggesting normalized levels of caspase activities and exosome secretion (Fig. 2).

### Plasma levels of miR-21, miR-27a and miR-218

**Fig. 3:**
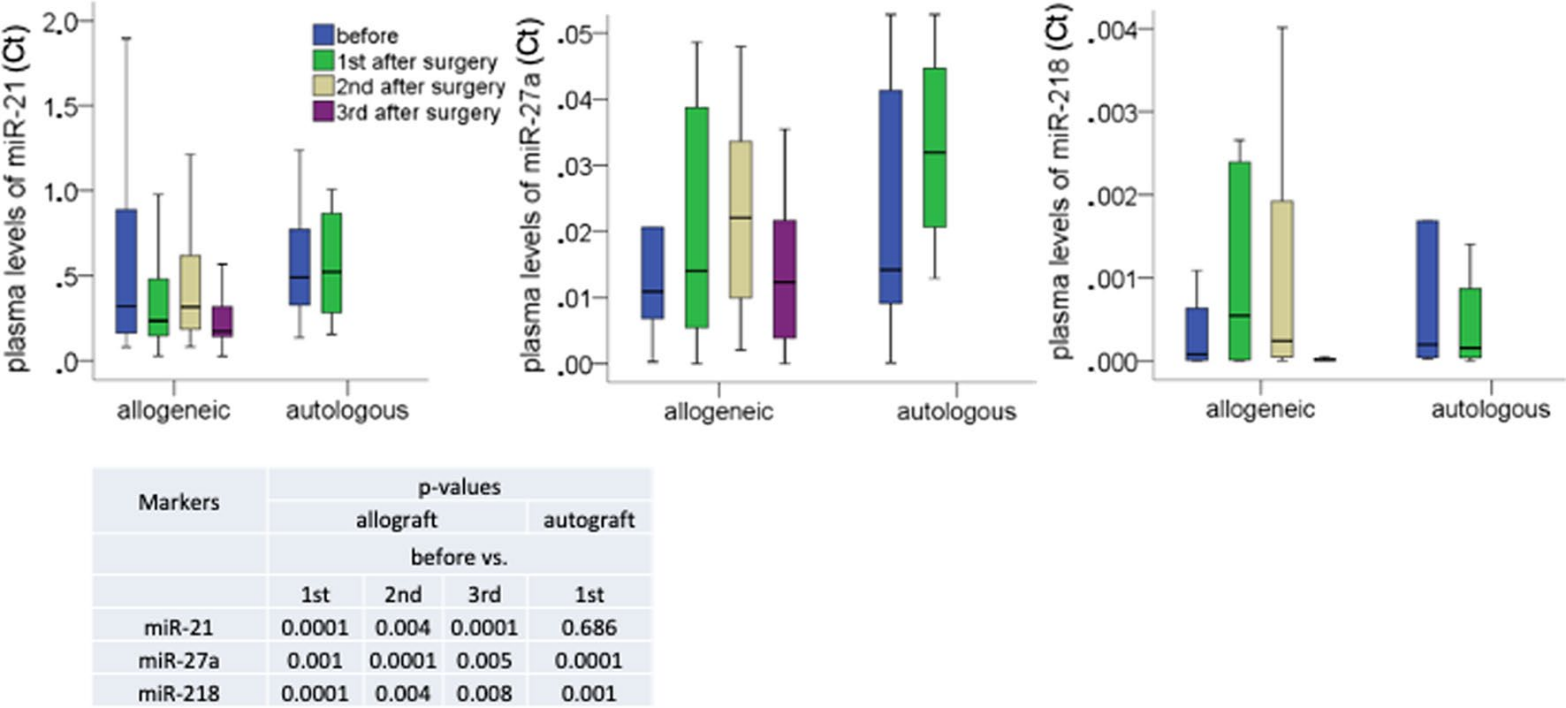
Deregulated plasma levels of miRNAs. The box blots show the plasma levels of miR-21, miR-27a and miR-218 in pre- and postsurgical plasma samples in 25 allogeneic and 10 autologous patients. MiRNAs were quantified by TaqMan PCR. Adjacent to the box plots, a table summarizes the p-values

In contrast to the above data, changes in the yields of specific miRNAs could be observed (Fig. 3). The levels of miR-27a and miR-218 significantly differed between the plasma samples before surgery and after surgery in both patient cohorts. The levels of miR-21 only significantly differed between the pre- and postsurgical plasma samples in the allogeneic cohort, but not in the autologous cohort. Strikingly, the levels of these miRNAs remained increased till 4 months after surgery (3^rd^ sample).

## Discussion

The focus of the present study was to analyze whether there are changes in the levels of circulating repetitive Alu sequences along their integrity, exosomes, caspase activities and miRNAs in the plasma of patients undergoing allogeneic and autologous intraoral bone grafting procedures.

Mesenchymal stem cells (MSC) play a major role in tissue regeneration and repair. Exosomes deriving from MSCs have a very high potential to regulate surrounding target cells via enzymes, cytokines, and growth factors [34]. Furthermore, exosomes can promote or suppress inflammatory activity in the neighboring cells by delivery of miRNA into those cells and therefore modulate the mRNA in these cells [35]. In order to further investigate those regulatory mechanisms during alveolar bone augmentation procedures in our present study, we quantified the changes in cfDNA levels, the cfDNA integrity of Alu247/Alu115 and caspase activities as well as the levels of exosomes and miRNAs in pre- and postsurgical plasma samples from patients who received either the aforementioned allograft or autologous graft. Quantification of the cfDNA levels of Alu115 and Alu247 indicates the source of cfDNA from apoptotic and necrotic cells, respectively, while the cfDNA integrity reflects the dimension of DNA fragmentation. Since caspases activate DNases and are involved in apoptosis, the measurement of their activities discloses the induction of apoptosis. However, when we statistically compared pre- and postsurgical plasma samples in both patient cohorts, we did not find any changes in cfDNA levels, fragmentation and cell death, namely apoptosis in both cohorts. Moreover, we only detected a significantly higher exosome levels before surgery and the first postsurgical sample in the autologous group than in the corresponding samples of the allogeneic group, but these significances may be caused by the high variance detected in the autologous group. However, exosomes contributing to cell communication play an important role in various physiological and pathological processes. ln dental diseases, they may be involved in anti-inflammatory and immunomodulatory effects as well as in tissue regeneration in oral regions, and repairing or reconstructing of bone grafts for implant and periodontal surgery [36]. Nevertheless, it was striking that the variance of the levels of caspase activities and exosomes decreased in the third postsurgical sample in the allogeneic cohort. Possibly, the broad variance reflects inflammatory parameters in some patients. Its decrease in the third postsurgical sample might cause similar normalized levels of caspase activities and exosomes in all patients of this cohort. Alterations in process of apoptotic signaling and thus, caspase activity were reported to occur in a vast range of oral diseases and surgical interventions [37]. The higher exosome and caspase levels before and in the first postsurgical sample might also be due to cytokines and chemokines, that are secreted in these inflammatory processes [38]. Possibly, the effect of medication might also play a role. The samples were taken on the day of surgery, and NSAIDs (nonsteroidal anti-inflammatory drugs) and other medicaments may affect the Na^+^/H^+^ exchanger or the Na^+^/Ca^2+^ channels and thus, the secretion of these molecules [39].

Our study shows that the levels of miR-27a and miR-218 significantly differed between the plasma samples before surgery and those after surgery in both patient cohorts. The levels of miR-21 only significantly differed between the pre- and postsurgical plasma samples in the allogeneic cohort, but not in the autologous cohort. Strikingly, the levels of all three miRNAs usually increased in the first or second postsurgical plasma sample, and decreased in the third postsurgical plasma sample, but were significantly elevated till 4 months after surgery. The continuous increase in miR-21 could be explained that miR-21 is a positive regulator of osteoblastic differentiation. Oka et al. [40] showed that miR-21 regulated osteogenic differentiation and mineralization by facilitating the expression of key osteogenic factors, such as alkaline phosphatase (ALP), runt-related transcription factor 2 (Runx2), Osteopontin (OPN), Osterix (OSX) and Mef2c in MC3T3-E1 cells. Therefore, miR-21 seems to play a major role in osteoblast differentiation and activation during bone regeneration and healing. Wei et al. [41] demonstrated that miR-21 levels were elevated during the regulation of osteogenic differentiation of periodontal ligament cells. These MSCs in dental and craniofacial tissues have a high potential for differentiation into osteoblasts. Diomede et al. showed that Exosomes derived from MSCs originating from gingival tissues and periodontal ligament cells promoted bone regeneration and repair, possibly by activating endogenous bone marrow MSCs [42]. This activation of MSCs can occur via cell communication through miRNA. Overexpression of miR-21 significantly inhibited osteogenesis of these MSCs, whereas its inhibition demonstrated opposite effects indicating that miR-21 may also be a key molecule during periodontal regeneration. Those findings strongly support our findings of a continuous increase of miR-21 during 4 months of healing following bone grafting. MiR-21 may also expose alveolar bone surgical stress and be associated with third molar postoperative pain onset [43]. In addition, the anti-inflammatory roles of miR-21 in lipopolysaccharide-stimulated human dental pulp cells were described [44, 45]. Thus, miR-21 seems to be a transcriptional modulator in dental tissues and bone regeneration. In their study, Wu et al. [46] demonstrated that overexpression of miR-27a positively regulated osteogenesis-angiogenesis coupling by inhibition of the pro-inflammatory cytokine TNF-α during bone formation in vitro. In addition, the same group showed that miR-27a had a strong impact during new bone formation and re-osseointegration of dental implants that lost bone support due to peri-implantitis. These findings suggest that miR-27a may play an important role in the maintenance and the stability of dental implants and support our results of an increase of miR-27a during the first 2 months of healing (2^nd^ blood sample after surgery) following bone grafting with an allogeneic bone grafting material where resorption of this material and bone osteogenesis-angiogenesis occurred. At a later point during the healing process, when the osteogenesis was completed, the level of miR-27a decreased again. In contrast, Chang et al. [47] identified miR-218 as a negative regulator of dentinogenesis of human dental pulp stem cells. Inhibition of miR-218 promoted the mineralization potentials of these cells after induction. In undifferentiated human-derived dental stem cells miR-218 targeted RUNX2, an osteogenic inducer, and decreased its expression. Consequently, mineralized tissue type differentiation was associated with a decrease in miR-218 expression controlling osteogenic differentiation of dental stem cells [48]. This finding is in conjunction with our findings showing a decrease in the expression of miR-218 during healing following bone augmentation with an allogeneic grafting material.

## Conclusion

In summary, within the limitations of this clinical trial due to the relatively low sample size, our previous findings as well as the results of the present study revealed very promising data. It should be considered that the samples were collected in a single center approach by one clinic only, in order to allow a better comparability of the data. Our findings indicate that there might be a high diagnostic potential in evaluating the plasma levels of the before mentioned miRNAs in order to detect bone resorption activities before they become clinically relevant and lead to advanced disease or even loss of alveolar bone, potentially resulting in loss of teeth or dental implants. Besides our analyses on circulating molecules and activities in plasma, the assessment of other factors, in particular growth factors, such as morphogenetic and inflammatory factors, as well as cytokines may improve intraoral bone grafting procedures [49].

Future studies on larger patient populations could further investigate if such a diagnostic test of plasma levels of molecular activities in combination with the clinical parameters could act as a liquid biopsy for the early detection, and therefore, prevention of intraoral destructive bone resorption processes. Especially in elderly multi-morbid patients, taking numerous medications influencing the equilibrium of bone remodeling processes, such an early diagnostic test could help to early diagnose and to prevent destructive processes around natural teeth and dental implants.

## Supplementary Information

**Additional file 1: Table S1**. This table shows the measured concentrations of miRNA 21, miRNA 27a, and miRNA 218 in relation to the different timepoints for the study popularion as well as for the control group.

**Additional file 2: Table S2**. This table shows the statistical analysis by unifactorial ANOVA analysis for Alu 115, Alu 247, Caspase, and Exosome measurements in relation to the different timepoints for the study popularion as well as for the control group.

## Data Availability

All data generated or analyzed during this study are included in this published article and its supplementary information files.
